# More Evidence for Teriparatide after Fixation of Atypical Femoral Fractures: Report of Four Cases

**DOI:** 10.5704/MOJ.2103.023

**Published:** 2021-03

**Authors:** WC Lee, THI Chua

**Affiliations:** Department of Orthopaedic Surgery, Tan Tock Seng Hospital, Singapore

**Keywords:** atypical femoral fracture, bisphosphonates, teriparatide

## Abstract

Atypical femoral fractures (AFF) have low union rates. The use of teriparatide has been advocated for the post-operative healing of AFF, but the evidence is limited to case reports and some series due to its low incidence.

We present a case series of four female patients to support the use of teriparatide after the surgical fixation of their AFF. Three of the patients had a complete AFF and one had an incomplete fracture. Their mean age was 70 (52 - 87) years, mean body mass index 24.6 (18.3 - 29.3), mean bone mineral density T-score of -2.3 (-4.8/-1.0), with a prior history of anti-resorptive therapy with bisphosphonates and denosumab.

Teriparatide was started at an average of 8 (2-18) days post-fixation, with 20mcg daily for six months. Immediate full weight-bearing was permitted in three patients, while one was non-weight bearing for two months. The mean time to union was 12 (10 - 14) weeks. No side effects were observed over a mean follow-up of 58 (50 – 72) weeks.

The use of teriparatide facilitated the quick union of AFF after surgical fixation. It appeared to be safe and promoted fracture healing in AFF.

## Introduction

Atypical femoral fracture (AFF) poses a challenge with high rates of delayed union over five months on average, and failure after fixation. The use of teriparatide has been advocated to improve the healing of surgically fixed AFF, but the evidence is limited^2^.

We present a case series of four patients with surgically fixed AFF, which united rapidly after being treated with teriparatide. This case series adds to the evidence for the post-operative use of teriparatide. We also discuss the optimal treatment dosage and duration.

## Case Report

Four patients who received teriparatide after surgical fixation of AFF were retrospectively identified. All patients met the American Society for Bone and Mineral Research (ASBMR) Task Force 2013 revised case definition for AFF^3^.

Table I shows the patients’ characteristics and treatment. After surgical fixation, the patients received daily subcutaneous injection of 20mcg of teriparatide for six months following consultation with endocrinologists. The patients were followed-up at the standard post-operative weeks 6, 12, 26, and 52 with radiographs upon review. Radiological union was defined as cortical continuity of three or more cortices on the anteroposterior and lateral views of plain radiographs.

**Table I:** Patient characteristics

	Patient 1	Patient 2	Patient 3	Patient 4	Mean
Age (years)	66	52	87	76	70
Gender	Female	Female	Female	Female	-
Bone mineral density (T-score)	-1.2	-1.0	-4.8	-2.0	-2.3
Complete or incomplete AFF	Complete	Complete	Complete	Incomplete	-
Prodromal symptoms	No	No	No	3 weeks	-
Pre-fracture supplementation with	Yes	No	Yes	Yes	-
calcium and vitamin D					
Serum vitamin D (μg/L)	32	18	20	29	25
Serum calcium (mmol/L)	2.31	2.38	2.22	2.39	2.33
Fixation method	Locking plate	IM nail with cerclage wiring	IM nail	IM nail	-
Post-operative weight bearing	NWB 8 weeks	Full	Full	Full	-
Teriparatide commencement	POD 6	POD 7	POD 18	POD 2	POD 8
Time to union (weeks)	11	14	14	10	12
Follow-up (weeks)	54	51	72	55	58

Abbreviations: AFF: Atypical femoral fracture, IM: Intramedullary, NWB: Non-weight bearing, POD: Post-operative day

Patient 1 sustained a midshaft fracture following a fall from a standing height. Patient 2 sustained a subtrochanteric fracture following a fall from a chair. Patient 3 sustained a midshaft fracture as she sat down due to “weakness”. Patient 4 had no trauma but had prodromal pain in the preceding three weeks before presentation. Although the fracture was incomplete, there were radiolucent lines laterally on the anteroposterior radiograph and anteriorly on the lateral radiograph.

No peri-surgical complications or adverse effects in relation to the use of teriparatide were noted.

## Discussion

Bisphosphonates are commonly used in the treatment of osteoporosis. It reduces the rate of bone turnover. The bone undergoes more mineralisation and is not substituted by younger, less mineralised bone, rendering it more intolerant to deforming forces and hence more brittle. AFF may occur, and this is a concern with the increasing use of bisphosphonates in the ageing population^2^.

With decreased bone turnover, a longer time to union, up to an average of 23 weeks, was observed after surgical fixation of AFF, despite halting any further use of bisphosphonates. A significant portion, up to 12%, would require revision within 48 weeks. For these, the union would occur only later, at an average of 44 weeks^1^.

The use of teriparatide to aid union has been described. Teriparatide is a recombinant form of parathyroid hormone (PTH) that causes increased bisphosphonates turnover and increases bone turnover. With its anabolic effect, the time to union and union rate after AFF have been reduced, in the few case reports and series. In Im and Lee's review, the largest series had 44 AFF in which Miyakoshi *et al* found that the union time was significantly reduced with teriparatide. Two other series reviewed only had 10 and 15 AFF^2^.

There is also no established dosage, frequency, and duration of treatment. In Im and Lee’s review, the interval from surgery to initiation of teriparatide ranged from 1 to 11 months, while the dose ranged from 20mcg daily to 56.5 mcg weekly, for a period of three months to two years^2^.

The use of teriparatide to aid union after surgical fixation of AFF is off the Food and Drug Administration (FDA) list of approved indications. Two dosing regimes have been described, either a daily subcutaneous injection of 20mcg daily or a weekly injection of 56.5mcg. Daily injection has been shown to achieve union in a significantly shorter time with an average of 26 weeks as compared to 44 weeks^3^. It should be commenced as soon as possible after the fixation as it has been shown to provide better radiographic healing as compared to a six months delay in therapy^4^. It is not definite when the therapy should cease, although the shortest reported duration is three months^2^.

All our cases united within 14 weeks. This was sooner than the mean 22 weeks reported by Bogdan *et al* Not all the patients received teriparatide in their study, and there was insufficient information on the treatment regime for those treated with it. These might be the reasons for the difference in our results^1^. Tsuchie *et al's* study showed a mean union time of 26 weeks. However, they included those treated non-surgically, which may increase the overall time to union^3^.

We also noted that Patient 4 achieved union within ten weeks, earlier than the other cases. A possible explanation for this is that teriparatide indeed has the potential to cause early union in all cases, and it became apparent when Patient 4 was coincidentally reviewed at an earlier date. A shorter interval for review for future cases may be required to determine this.

None of our patients developed adverse effects from the treatment. Nevertheless, physicians need to be cognisant of the potential adverse effect of hypercalcaemia which may occur in up to 11% of patients. Non-uraemic calciphylaxis has been reported, albeit extremely rare. There is also a theoretical risk of osteosarcoma, though it was only noted in rat studies and may be dose and duration-dependent. Hence, it might be desirable to stop the treatment once the fracture is united. Nonetheless, this needs to be balanced against the risk of future fracture, as the bone density undergoes accelerated deterioration upon cessation of teriparatide. Ordinarily, in osteoporosis, this may be mitigated by taking other anti-osteoporotic medications such as bisphosphonates or denosumab, but this may not be suitable for patients who already had AFF^5^.

In conclusion, current literature provides encouraging results for the use of teriparatide after the surgical fixation of AFF. It is difficult to perform a study with a high level of evidence due to its rare occurrence. Nevertheless, each series adds to our understanding of the utility of teriparatide in improving the union in surgical fixation of AFF. We have described in detail our treatment regime and future studies should also include this to guide clinical management.

## References

[1] Bogdan Y, Tornetta P, Einhorn TA, Guy P, Leveille L, Robinson J (2016). Healing Time and Complications in Operatively Treated Atypical Femur Fractures Associated With Bisphosphonate Use: A Multicenter Retrospective Cohort.. J Orthop Trauma..

[2] Im GI, Lee SH (2015). Effect of Teriparatide on Healing of Atypical Femoral Fractures: A Systemic Review.. J Bone Metab..

[3] Tsuchie H, Miyakoshi N, Iba K, Kasukawa Y, Nozaka K, Dohke T (2018). The effects of teriparatide on acceleration of bone healing following atypical femoral fracture: comparison between daily and weekly administration.. Osteoporos Int..

[4] Starr J, Tay YKD, Shane E (2018). Current Understanding of Epidemiology, Pathophysiology, and Management of Atypical Femur Fractures.. Curr Osteoporos Rep..

[5] Eastell R, Walsh JS (2017). Anabolic treatment for osteoporosis: teriparatide.. Clin Cases Miner Bone Metab..

